# Update on Chitosan-Based Hydrogels: Preparation, Characterization, and Its Antimicrobial and Antibiofilm Applications

**DOI:** 10.3390/gels9010035

**Published:** 2022-12-30

**Authors:** Kokila Thirupathi, Chaitany Jayaprakash Raorane, Vanaraj Ramkumar, Selvakumari Ulagesan, Madhappan Santhamoorthy, Vinit Raj, Gopal Shankar Krishnakumar, Thi Tuong Vy Phan, Seong-Cheol Kim

**Affiliations:** 1 Department of Physics, Sri Moogambigai College of Arts and Science for Women, Palacode 636808, India; 2 School of Chemical Engineering, Yeungnam University, Gyeongsan 38541, Republic of Korea; 3 Division of Fisheries Life Sciences, Pukyong National University, Nam-gu, Busan 48513, Republic of Korea; 4 Department of Biotechnology, Applied Biomaterials Laboratory, PSG Institute of Advanced Studies, Coimbatore 641004, India; 5 Center for Advanced Chemistry, Institute of Research and Development, Duy Tan University, 03 Quang Trung, Hai Chau, Danang 550000, Vietnam; 6 Faculty of Environmental and Chemical Engineering, Duy Tan University, 03 Quang Trung, Hai Chau, Danang 550000, Vietnam

**Keywords:** chitosan-based hydrogel, structural characterization, antibacterial activity, antifungal materials, antibiofilm

## Abstract

Chitosan is a prominent biopolymer in research for of its physicochemical properties and uses. Each year, the number of publications based on chitosan and its derivatives increases. Because of its comprehensive biological properties, including antibacterial, antioxidant, and tissue regeneration activities, chitosan and its derivatives can be used to prevent and treat soft tissue diseases. Furthermore, chitosan can be employed as a nanocarrier for therapeutic drug delivery. In this review, we will first discuss chitosan and chitosan-based hydrogel polymers. The structure, functionality, and physicochemical characteristics of chitosan-based hydrogels are addressed. Second, a variety of characterization approaches were used to analyze and validate the physicochemical characteristics of chitosan-based hydrogel materials. Finally, we discuss the antibacterial, antibiofilm, and antifungal uses of supramolecular chitosan-based hydrogels. This review study can be used as a base for future research into the production of various types of chitosan-based hydrogels in the antibacterial and antifungal fields.

## 1. Introduction

Biopolymers are crucial in biological applications due to their biocompatibility and biodegradability. Biopolymer-based materials are frequently regarded as viable materials for delivering various therapeutic agents due to their improved biodegradation and biodistribution in biological systems. Researchers are interested in a variety of biopolymers, including chitosan, alginate, pectin, cellulose, agarose, and gelatin [1,2,3]. Chitosan is a naturally occurring cationic polymer produced by chitin deacetylation. Chitosan is a co-polymer of D-glucosamine and N-acetyl-D-glucosamine. The amount of D-glucosamine and N-acetyl-D-glucosamine residues in the co-polymer is affected by the degree of deacetylation. It has been widely used in healthcare due to its potent antibacterial and antifungal effects. The different physical and chemical properties are due to the many chemical structures.

**Scheme 1:**
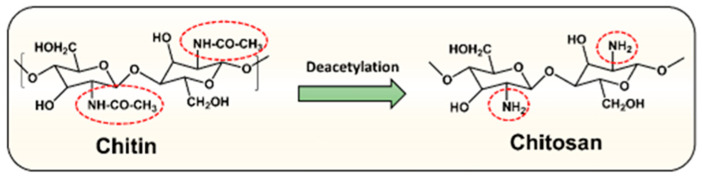
Chemical conversion of chitin onto chitosan by deacetylation process.

Chitosan solubility is influenced by a range of parameters, including polymer molecular weight, degree of acetylation, pH, temperature, and polymer crystallinity [4]. The chitosan solubility depends on the amount of acetylation and deacetylation process. The low solubility of deacetylated chitosan was attributed to an increase in polymer crystallinity. In contrast, a reduction in the crystallinity was observed in the half-acetylated samples. These derivatives were developed by chemical modifications that preserved the original chitosan’s beneficial biological features while improving its physical and chemical properties [5]. Chitin is a widespread biopolymer found in crustaceans, insect cuticles, algae, and fungus cell walls, whereas chitosan is only found in a few fungi in nature. Most commercial chitosan obtained by chemical deacetylation of crab chitin [6]. Vegan requirements have driven recent commercial interest in chitosan derived from fungus. Furthermore, these samples have a very high degree of deacetylation and are carefully controlled in terms of low viscosity. Chitin is a natural biomacromolecule material found in lower creature shells, notably arthropod shells, as well as lower plant, bacterium, and algae cell walls. Because of their low cost and high chitin concentration, shrimp/crab shells are currently used as the primary raw material for manufacturing chitin [7]. Chitosan is a deacetylated chitin derivative containing at least 50% free amine (Scheme 1). Chitin is abundant, has a unique structure, and has excellent biological properties. It may be produced into gels, fibers, films, and microspheres, among other things, and it can also be used as a raw material to make other materials [8].

**Scheme 2:**
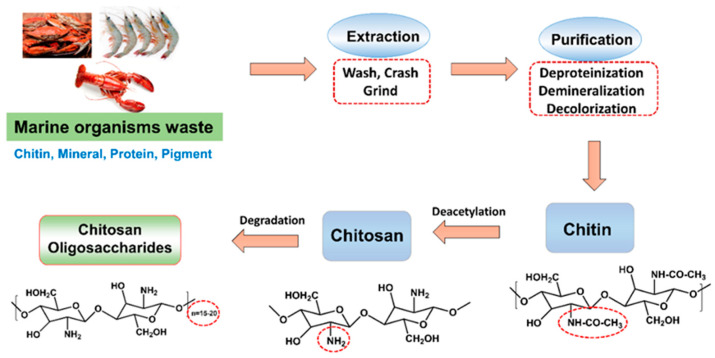
Outline of production and chemical structure of chitosan.

Currently, chitosan is mostly derived from shrimp and crab shells, which are abundant in resources, give a high output, and are inexpensive (Scheme 2). However, there are two drawbacks to this method: first, the collection of chitosan is seasonal and expensive, and second, the quality and quantity of chitosan are not consistent owing to varying types of raw materials, manufacturing sites, and production procedures. Since the 1990s, researchers have been investigating the fermentation technique of manufacturing chitosan [9]. This technology can significantly lower chitosan manufacturing costs and allow for the replacement of the shrimp shell or crab shell as the raw material. Chitosan is the only polycation found in nature, and its charge density varies depending on the degree of acetylation and the pH of the medium. The degree of acetylation and the molecular weight of the polymer impact its solubility. Chitosan oligomers are soluble at all pH levels, from acidic to basic. Higher molecular weight chitosan samples, on the other hand, are exclusively soluble in acidic aqueous conditions, even at high deacetylation levels. Because chitosan is difficult to dissolve at neutral and basic pH levels, its usage in a range of applications under neutral pH circumstances has been limited [10].

**Scheme 3:**
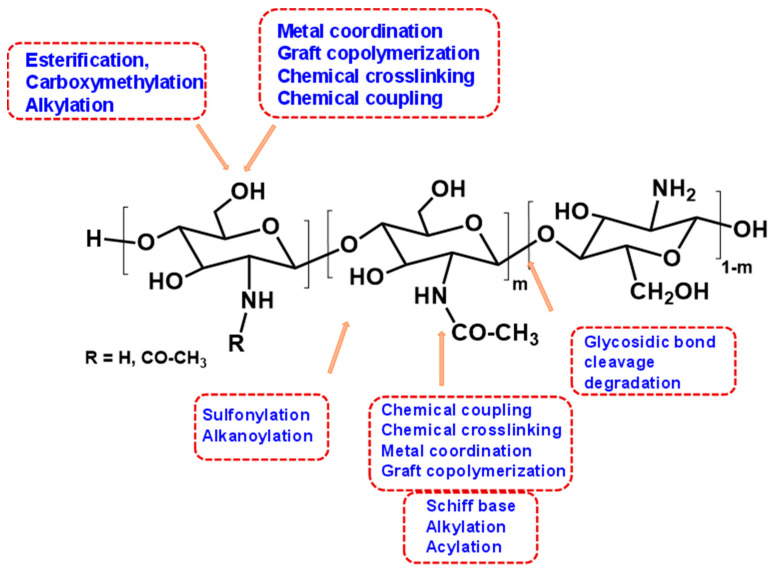
Functional groups in chitosan polymer that can be chemically modified. M: monomer units.

The solid morphology of chitosan is maintained through the intermolecular and intramolecular interactions. Chitosan is more reactive because the macromolecular chain generated by deacetylation of chitin contains amino groups. The presence of amino groups allows for a wide range of chemical reactions, such as quaternization, alkylation, and metal chelation [11]. Chitosan dissolves exclusively in acidic or aqueous solutions including LiOH/KOH/urea. The breaking of intramolecular and intermolecular hydrogen bonds in chitosan was mostly caused by LiOH [12]. This greatly limits its usefulness. Chemical alteration of chitosan chains, such as the addition of hydroxyalkyl, carboxyalkyl, acyl, and other hydrophilic groups, can significantly enhance water solubility (Scheme 3). N-succinyl chitosan (NSCS) is a water-soluble chitosan derivative with enhanced water retention [13]. As glucosamine is a basic unit of chitosan in which many active amino and hydroxyl groups are present. Hence, chitosan can be distinctly modified by acylation, carboxylation, and etherification to produce a series of chitosan derivatives with varying properties, which can improve its water solubility, biological activity, and mechanical properties and broaden chitosan’s application. Modified chitosan derivatives are gaining popularity because they outperform unmodified chitosan in chemical, biological, and functional characteristics such as solubility and gelation.

Hydrogels are built with a distinctive three-dimensional (3D) hydrophilic polymeric network which can absorb large amounts of aqueous and biological fluids without undergoing any dissolution. This unique characteristic property of hydrogels is often compared with natural tissues/organs owing to their same degree of flexibility which can be exploited for a broad spectrum of biomedical applications [14]. The inception of hydrogels in biological applications antecedes the 19th century when they were mainly colloidal gels derived from inorganic salts. Hydrogels are also primitive biomaterials to be used in human body implantation. Generally, hydrogels are derived from both natural and synthetic materials [15]. For instance, natural polysaccharides, such as alginate, dextran, and chitosan and proteins, such as collagen, gelatin, silk, and fibrin, are examples of natural hydrogels that have been used extensively in various biomedical applications owing to their excellent biocompatibility, biodegradability, and biosorption. Likewise, synthetic hydrogels are commonly derived from poly(vinyl alcohol) (PVA), polyethylene oxide (PEO), and poly(acrylic acid) (PAA) due to their advantageous properties, such as high chemical purity, reproducibility, controlled degradation, good mechanical properties, and low cost [16].

Hydrogels are categorized into different generations based on their synthesis and applications. The first-generation hydrogels were simple polymeric network hydrogels without any supplementary features. The second-generation hydrogels were stimuli-responsive (pH/temperature) hydrogels that could respond to changes in environmental conditions. The third-generation hydrogels were stererocomplexed hydrogels, where cross-linking mechanisms were used to improve the physical properties. Finally, the fourth-generation hydrogels are contemporary hydrogels commonly referred to as smart hydrogels, which are tailored composite hydrogels with double networking structures synthesized through a radical polymerization process to improve the overall physicochemical and biological properties [17]. Owing to the extensive scientific research in hydrogels and rapid advancements in synthetic chemistry, there has been a development of smart hydrogel systems to meet specific needs depending on their applications. Undoubtedly, hydrogels are the mainstay choice of materials in a myriad of applications, such as drug delivery, wound healing, antibacterial coatings, 3D bioprinting, and cell encapsulation [18].

Worldwide, microbial infections pose a significant threat and challenge to the public health system. Furthermore, the increase in global antimicrobial resistance has shown dire consequences causing a huge economic burden. Among the several potential candidates for alternative antimicrobials, hydrogels with organized intermolecular self-assembling structures are gaining increasing interest in many therapeutic branches of medicine [19,20]. The intrinsic ability of hydrogels to restrain bacterial infection stems from the inherent mechanism of action. Unlike antibiotics, hydrogels, instead of acting on the intracellular sites, target the microbial membrane thereby reducing the possibility of a microbe developing resistance. Furthermore, hydrogels exhibit programmable antimicrobial capability and adjustable mechanical strength with a good degree of biocompatibility [21]. Antimicrobial hydrogels are extremely alluring materials often developed either by (i) loading them with pharmaceutical recipients (drugs) or (ii) covalently ligating active agents by self-assembly mechanisms. In the first method, hydrogels serve as a carrier system providing a controlled release of bioactive molecules, such as proteins, peptides, nanoparticles, and nucleic acids with antimicrobial benefits [22]. The non-covalent encapsulation of pharmaceutical ingredients within the gel network structure will allow a slow and sustained release of therapeutic compounds, which imparts adequate antimicrobial functions at the target site. In the second method, inherently active hydrogels can be produced by the self-assembling process, where monomers are synthesized initially and allowed to assemble into hydrogel networks with covalent ligation of active agents, which can exhibit antimicrobial activity [23].

Hydrogels are water-saturated polymeric substances with three-dimensional network structures that are composed of polymer chains crosslinked through physical or covalent bonds [24,25]. Hydrogels are generally hydrophilic in nature. Due to the hydrophilic functional groups, hydrogels may expand in water by absorbing tens or hundreds of times more water than their own mass. The hydrogels can be used for wound healing because of their biocompatibility and cell adhesion properties [26]. Hydrogels have gained major attention due to their high water content, softness, flexibility, and ability towards many biomedical applications [27]. Bacteria become resistant due to the overuse of antibiotics. To address this issue, antibacterial hydrogels, having hydrogel and antibacterial activity, have been developed. So far, a variety of antibacterial hydrogels has been developed in response to their simple preparation and diverse structure, which attracts huge attention.

## 2. Preparation of Various Chitosan-Based Hydrogels

### 2.1. Classifications and Characterization Techniques of Hydrogels

Chitosan-based hydrogels derived from both synthetic and natural polysaccharides are ideal scaffolds for use in biomedical applications. In particular, polysaccharide-based materials are highly attractive in nanomedicine as they are nontoxic, abundant, and often bioactive due to their structural similarities with the extracellular matrix and have, therefore, found applications in various biomedical fields. Furthermore, chitosan, a natural biopolymer-based hydrogel, is considered to be a promising material for various biological applications owing to its bioactivity, biocompatibility, and biodegradability. The hydrogels consist of cross-linked polymer chains of dimensions ranging from 100 nm to several micrometer, which can swell in a good solvent while retaining their initial three-dimensional structure [28]. These nanomaterials have been extensively exploited for the encapsulation and controlled release of drugs or biological molecules. 

Hydrogels are generally classified as physical hydrogels and chemical hydrogels.

### 2.2. Physical Hydrogels

Physical techniques include noncovalent, reversible links or interactions between polymer chains, such as ionic, electrostatic, and hydrophobic interactions, grafting, and entanglement [29]. pH, polymer concentration, and temperature are among the physical parameters that can impact hydrogel formation. Furthermore, the number of chemical contacts between reacting molecules dictates hydrogel integrity; hence, enhanced interactions result in stiff hydrogels, whereas restricted connections result in a soft, weak hydrogel structure. Polyelectrolyte-complexed hydrogels are physically manufactured hydrogels created by ionic interactions between their functional component polymers. Physical hydrogels are created by combining two polyelectrolytes in solution with opposing charges. Chitosan-based hydrogels are generated by the electrostatic interaction of polymer positive or negative functionalities with chitosan amine units.

### 2.3. Chemical Hydrogels

Chemical hydrogels are created by covalently bonding polymers together through chemical reactions. The need for chemical modification of the chitosan structure, as well as their irreversible nature, distinguishes these hydrogels. Amide and ester bonding, as well as Schiff base, are examples of links that can occur during chemical hydrogel production. A crosslinker is required to create chitosan-based hydrogels. Crosslinkers, such ethylene glycol, diglycidyl ether, and others, are frequently needed to react with previously activated functional groups in the chitosan polymeric chain [29]. Purification of chemical hydrogels is often necessary following the hydrogel elaboration procedure to eliminate unreacted crosslinkers. The cross-linking density and crosslinker ratio are the structural dictating elements in hydrogel production.

Numerous common characterization approaches are utilized to characterize hydrogels. Depending on the hydrogel qualities and application, several characterizations are utilized. Rheological properties, as well as absorption and degradation potential, are crucial considerations. Overall, the hydrogel morphology, swelling, and mechanical resistance are all crucial factors to consider. Chitosan-based hydrogels frequently required property measurement and structural investigation. Spectroscopic and microscopic methods were mostly employed in structural investigation. Furthermore, the performance attributes of hydrogels are varied by their specific uses.

## 3. Various Approaches on the Preparation of Chitosan-Based Hydrogels

Hydrogels are soft and three-dimensional network polymers and their intrinsic characteristic is cross-linking, which exists in those materials. The cross-linking density makes a critical difference in dominating the performance of hydrogels. Currently, there are several methods available for the synthesis of chitosan-based hydrogel, which includes physical interaction and chemical cross-linking. Furthermore, chemical cross-linking is permanent and the structure is very stable, while physical cross-linking is dynamic and the structure is highly dependent on external environments.

### 3.1. The Preparation of Hydrogels by Physical Interaction

#### 3.1.1. The Natural Product’s Materials’ Physical Composition

**Figure 1:**
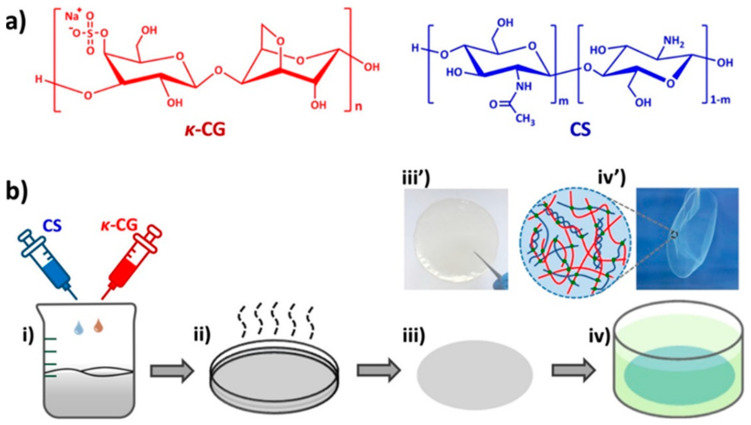
(**a**) Chemical structure of kappa-carrageenan (k-CG) and chitosan (CS). (**b**) The schematic representation on the preparation of ultra-thin κ-carrageenan/chitosan hydrogel films. Reprinted with permission from [30] Copyright 2018 American Chemical Society.

The development of hydrogel utilizing natural product components is seen as a promising strategy. Yu and colleagues used this technique to create carrageenan- and chitosan-based hydrogel for food packaging applications [30]. The varied weight percentages of k-carrageenan and chitosan were dissolved in acetic acid while vigorously stirring, then transferred to a Petri dish and maintained at 70 °C. The transparent thin film-like layer was removed and used for further investigation (Figure 1). This technique resulted in polysaccharide-based hydrogel films with outstanding mechanical capabilities and antiadhesion qualities, and the resultant hydrogel film has a thin sheet with high toughness and antiadhesion properties. The developed -CG/CS gel films had good mechanical characteristics with a breaking stress of 2–6.7 MPa and a breaking strain of 80–120%, which were superior to most existing biopolymer-based hydrogels. They found that the exceptional mechanical properties of gel films obtained over a wide range of k-CG-to-CS mass ratios due to the synergistic impact of ionic and hydrogen interactions between the -CG and CS molecules.

#### 3.1.2. The Nanocomposite-Based Hydrogels Preparation

**Figure 2:**
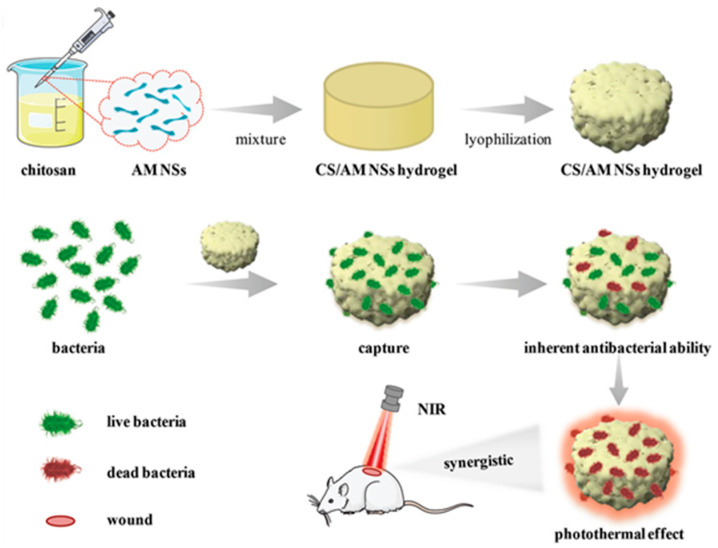
The schematic representation of the preparation of CS/AM NSs hydrogel and its application towards the bacterial wound treatment on the mice, adopted from [31] with permission from Wiley through copyright clearance center.

The physical interaction between the chitosan and some materials leads to the formation of hydrogel in nature; this type involves the surface elongation between the molecules without chemical reaction. Liu et al. reported the chitosan and antimonane nanosheets based hydrogels for the antibacterial applications (Figure 2). The hydrogels were prepared by bidirectional freeze casting and lyophilization techniques [31]. 

**Figure 3:**
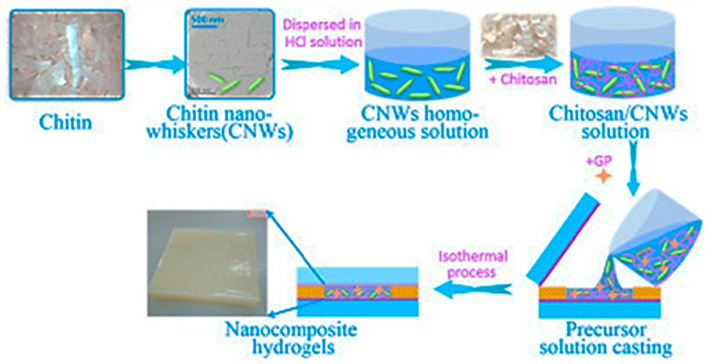
The graphic representation on the preparation of CNWs/CS nanocomposites, adopted from [32] with permission from Elsevier through copyright clearance center.

Wang et al. [32] prepared chitosan and chitin nano-whiskers (CNWs)-based nanocomposites hydrogel for the tissue engineering solicitations. The CWNs were mixed with chitosan/β-glycerophosphate disodium salt (CS/GP) to obtain a targeted injectable hydrogel material (Figure 3).

#### 3.1.3. The Polymer Composite-Based Hydrogels Preparation Method

**Figure 4:**
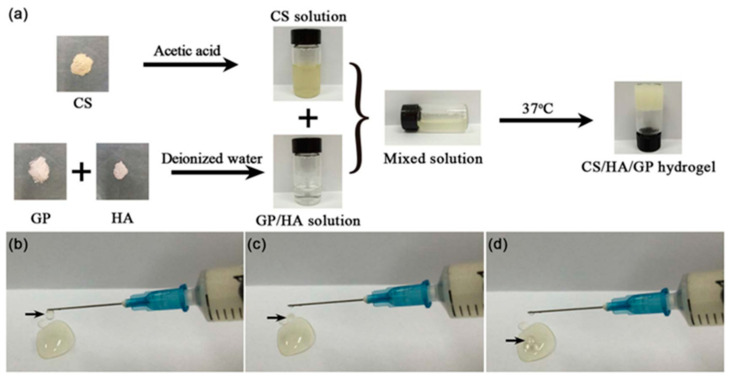
(**a**) The preparation of hydrogel precursor solution (HG3) and the solution at low temperature of 4 °C and the formed hydrogel at 37 °C, (**b**–**d**) the HG3 precursor solution can be readily ejected to form droplets using a syringe, adopted from [33] with permission from Elsevier through copyright clearance center. CS: Chitosan; HA: Hyaluronic acid; GP: Glycerophosphate.

Similarly, the injectable and body-temperature-sensitive hydrogel were prepared by Zhang et al. (Figure 4). The chitosan, hyaluronic acid and /β-sodium-glycerophosphate-based hydrogels were prepared by physical mixing at room temperature. The proposed work is highly applicable in temperature- and pH-sensitive drug release applications [33].

#### 3.1.4. The Preparation of Thermosensitive Hydrogel

**Figure 5:**
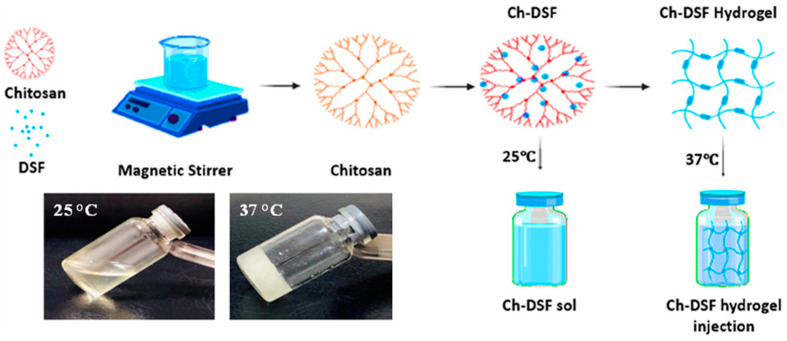
The schematic representation of the preparation of chitosan-based thermosensitive hydrogels. Insert: the hydrogel at room and body temperature, adopted from open access resource [34]. DSF: disulfiram.

The thermosensitive hydrogel materials are crucial for biological applications. These materials have the unique property of being solid at high temperatures and liquid at low temperatures. By considering this aspect, Ashan et al. [34] demonstrated the use of chitosan- and disulfiram-based hydrogels for anticancer drug delivery applications (Figure 5). Chitosan and hyaluronic acid were dissolved in water and agitated for two hours to create this hydrogel. After that, the mixture was ultrasonicated for 30 min to create the necessary hydrogel composition. These hydrogels demonstrated high biocompatibility after fast gel formation at body temperature. The swelling index and in vitro drug release assays revealed that the hydrogel produced is pH-sensitive and has selective drug release capabilities.

**Figure 6:**
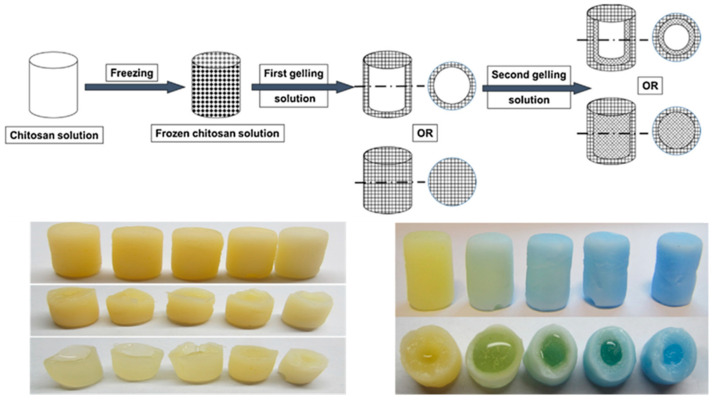
The schematic illustration of the preparation of the chitosan-based hydrogel; insert: photo images of the obtained hydrogel materials, adopted from [35] with permission from Elsevier through copyright clearance center.

In this regard, Xu et al. [35] used a step-by-step cross-linking approach using sodium hydroxide and sodium tri-phosphate cross-linkers to create physically cross-linked chitosan hydrogels with gradient structures (Figure 6). By varying the gelation procedure, single-, double-, and triple-layered hydrogel structures were created. To analyze the cross-linking of hydrogels, several factors, such as pH, gelation time, gelation conditions, and compositions, were used. 

#### 3.1.5. Polymer Nanocomposite-Based Hydrogels Preparation

**Figure 7:**
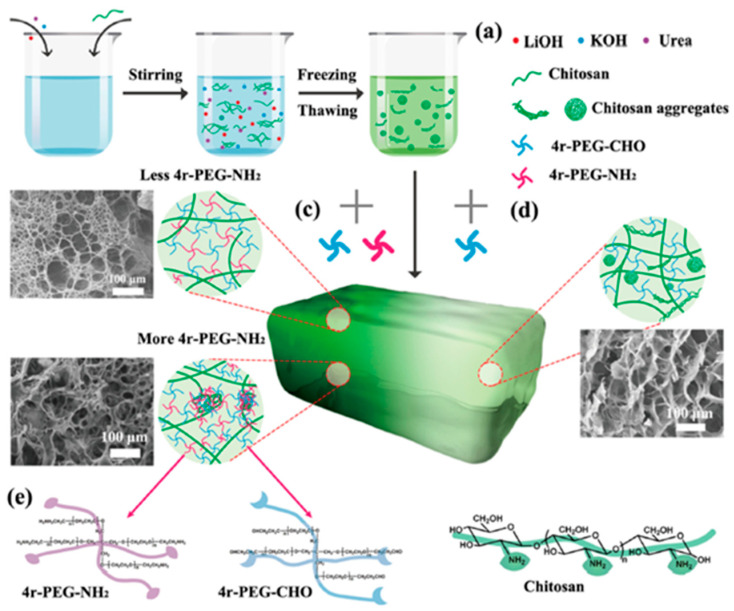
The schematic representation with respect to the synthesis and structure of Cn nm gels. (**a**) The dissolution process of chitosan in alkaline solution; (**c**) The structure of gels with only 4r-PEG-CHO; (**d**) The structure of gels with 4r-PEG-CHO and 4r-PEG-NH2; (**e**) The molecular structure of the reactants. The SEM image on the right belongs to C2-N0 gel, the SEM images on the top left and bottom left belong to C2-N2 gel and C2-N4 gel, respectively, adopted from [36] with permission from Elsevier through copyright clearance center. PEG: Polyethyleneglycol.

Liu et al. created a chitosan-based hydrogel network by employing macromolecule aldehyde-four-arm polyethylene glycol as a cross-linker via the Schiff base reaction between chitosan’s aldehyde and amine groups. The resulting hydrogels were used for antibacterial wound treatment (Figure 7). The scaffold development to mimic the gradient structure of natural tissue is an important consideration for effective tissue engineering [36]. 

### 3.2. Preparation of Hydrogels Using Chemical Reaction 

#### 3.2.1. The Hydrogels Prepared from Condensation Method

**Figure 8:**
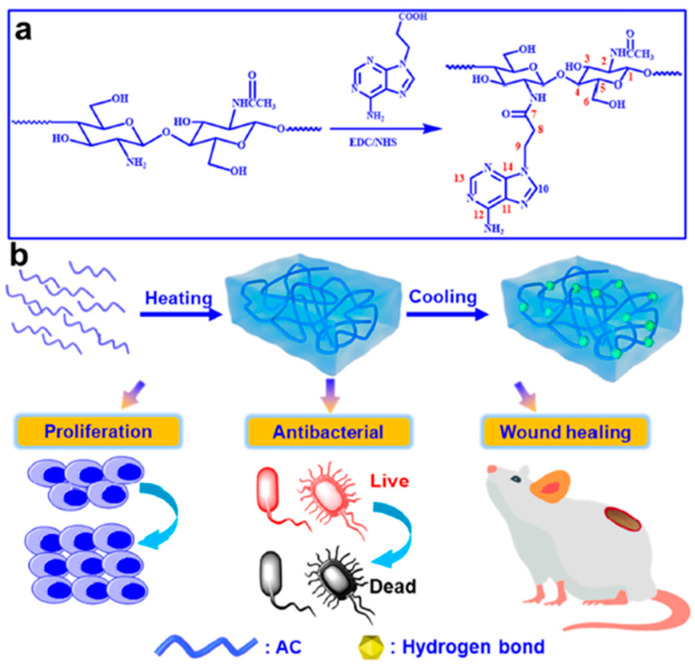
(**a**) The synthetic route of AC derivatives. (**b**) Schematic diagram of AC hydrogel preparation and promotion of wound healing, adopted from [37] with permission from Elsevier through copyright clearance center. AC: Adenine-modified Chitosan.

The hydrogels are formed as a result of chemical (covalent) bonding between chitosan and other materials. Deng and coworkers [37] used an amidation reaction technique to create an injectable adenine-modified chitosan derived by reacting the chitosan amine groups with carboxyl adenine groups (Figure 8a,b). The self-healing and wound healing capabilities of the produced chitosan-based hydrogels demonstrated that the prepared hydrogel could be efficient in antibacterial wound healing applications. 

**Figure 9:**
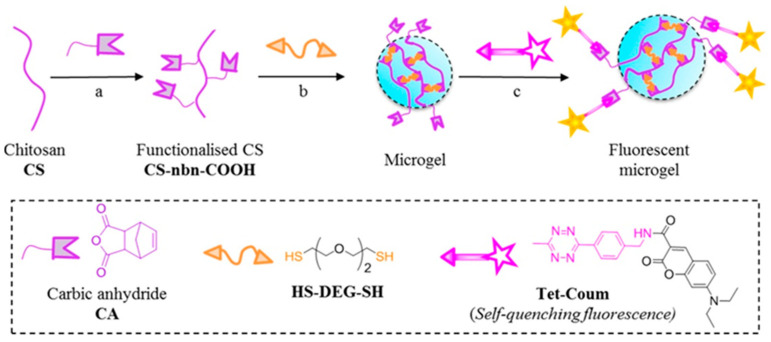
Synthesis of norbornene-derived chitosan-based microgels, reprinted with permission from [38]. Copyright 2019 American Chemical Society.

The chitosan-based nanocomposites are efficient methods in the preparation of hydrogel materials for the multipurpose applications. Sarah et al. [38] prepared the chitosan- and poly(N-isopropylacrylamide) [p(NIPAAm)]-based nanohydrogels for the bio-medical applications (Figure 9). The synthetic method involves through the comprising and cross-linking process. In this aspect, the preparation of norbornene-derived chitosan-based microgel under the rapid gelation method by using thiolated crosslinker via photo-induced thiol–ene click chemistry. In this method, the natural chitosan and commercially available carbic anhydride were used for this hydrogel preparation via a single-step process. The proposed synthetic approach would pave the path to develop various types of hydrogels for the development of appropriate biomedical devises.

#### 3.2.2. The Hydrogels Synthesized from the Ring-Opening Method

**Figure 10:**
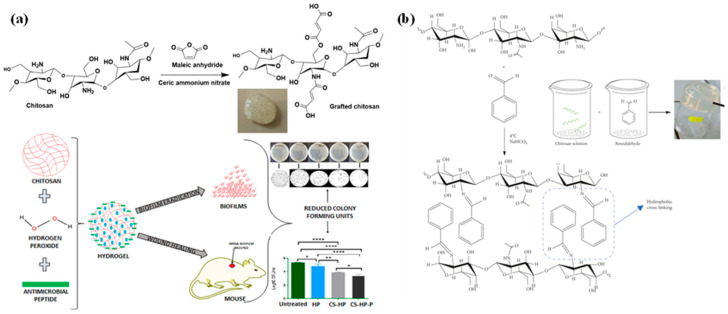
(**a**) Chitosan-based hydrogel preparation using maleic anhydride towards anti-microbial applications, (**b**) the preparation of chitosan-based hydrogels using benzaldehyde using lyophilization method, adopted from open access resource [39,40].

The chitosan polymer can be cross-linked using small molecules as a cross-liner, which can react with amine groups of chitosan units. For example, the chitosan could be cross-linked with maleic anhydride and benzaldehyde (Figure 10a). Fasiku and colleagues [39] described the integration of maleic anhydride into chitosan and maleic anhydride-based hydrogels for antibacterial applications. The synthesized hydrogel exhibited antibacterial characteristics and was used for dual antibiotic medication delivery and wound-healing applications. Damiri et al. [40] created a chitosan and benzaldehyde-based hydrogel for drug delivery (Figure 10b). The lyophilization procedure was employed in this study to prepare the desired hydrogel substance. In a similar study, Liu et al. [41] used the Schiff base reaction between chitosan and konjac glucomannan to create injectable self-healing hydrogels. The produced hydrogels showed outstanding antibacterial and wound healing characteristics.

#### 3.2.3. The Hydrogels Produced from the Cross-Linking Method

**Figure 11:**
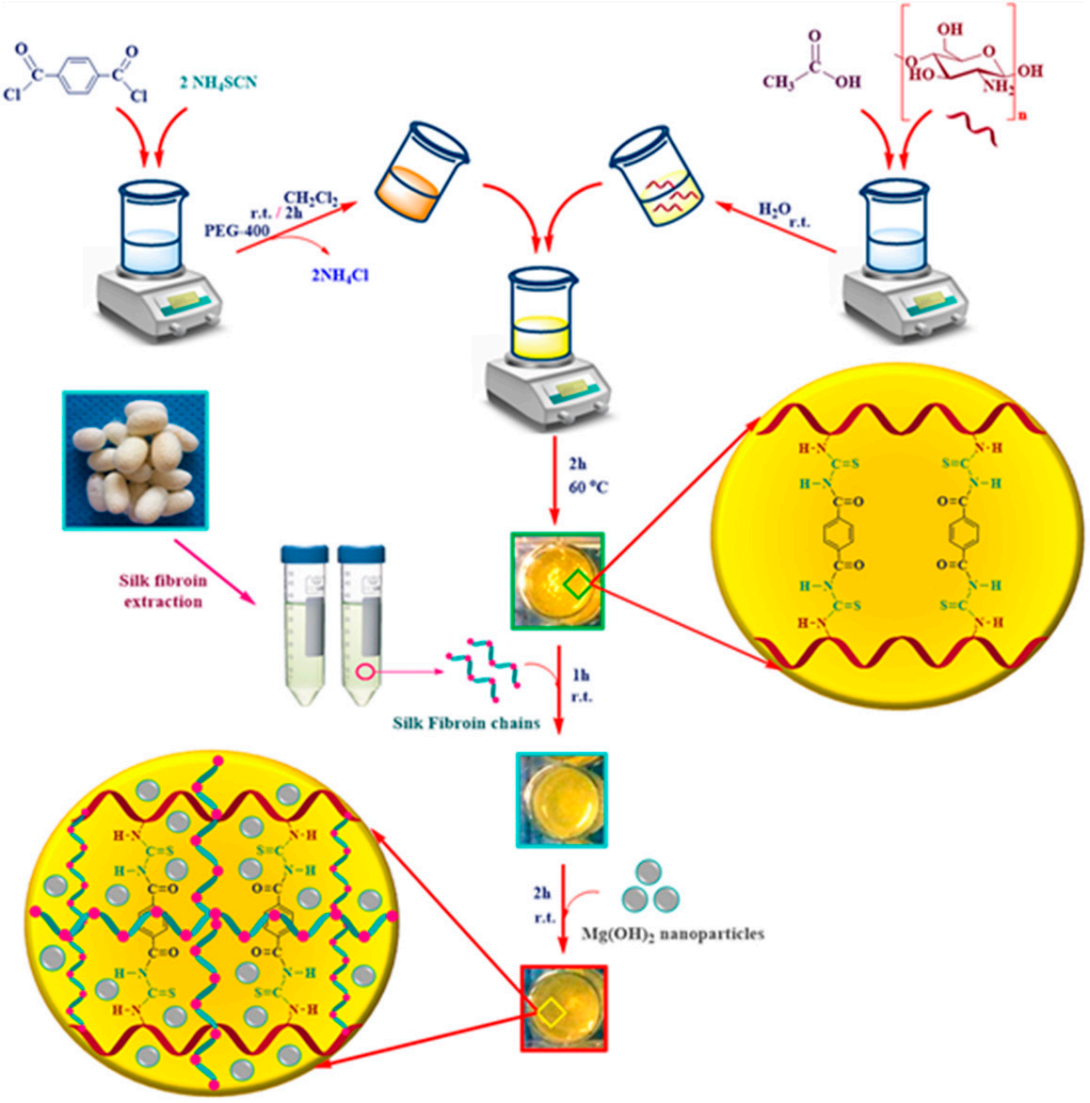
The schematic depiction on the preparation of chitosan hydrogel nanocomposites, adopted from open access resource [42].

The present work reports a novel nanobiocomposite support based on modifying synthesized cross-linked terephthaloyl thiourea–chitosan hydrogel (CTT-CS hydrogel) substrate followed by the extracted silk fibroin (SF) biopolymer and the incorporation of Mg(OH)_2_ nanoparticles (Figure 11). The prepared materials were applied towards the anti-bacterial and anti-fungal applications [42].

## 4. Characterization Techniques of Chitosan Hydrogels

### 4.1. Structural Analysis

The investigation of the microstructural properties that may have an influence on the structural integrity of hydrogels. For direct imaging of chitosan hydrogel, microscopic methods, such as scanning electron microscopy (SEM) and transmission electron microscopy (TEM) are commonly used. SEM is used to evaluate the surface morphology of various forms of the hydrogel matrix. Hydrogel samples are often covered with a thin layer under vacuum for SEM imaging studies. TEM allows for a full examination of hydrogel samples, including changes in chemical composition, orientation, and aspect ratio (length-to-diameter or length-to-width ratio) of nanostructures, as well as the induction of electronic phase shifts and images based on material absorption. These findings support the argument over the influence of different therapies, as well as the optimization of hydrogel preparation conditions [43].

**Figure 12:**
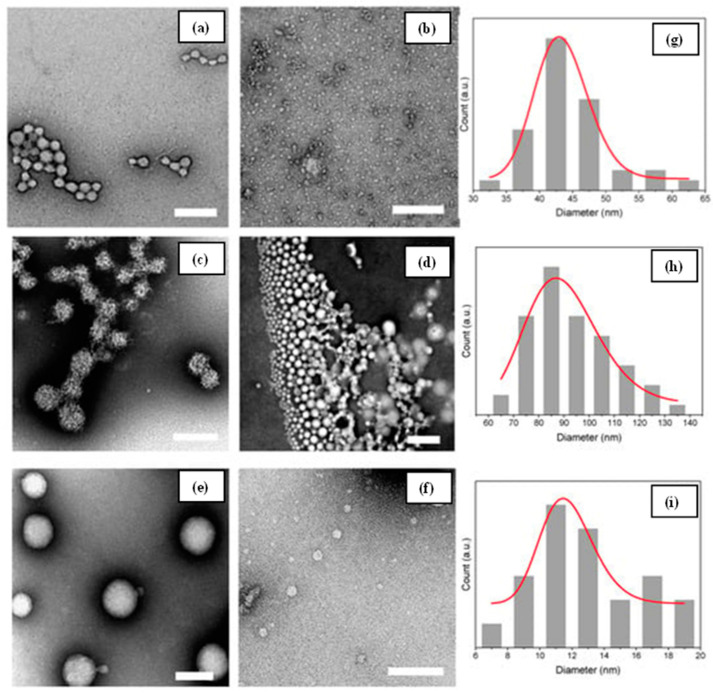
TEM images of prepared chitosan caseinate PEC nanoparticles (**a**–**f**), and the DLS particle size analyses results (**g**–**i**), adopted from open access resource [44].

Abdalla et al. [45] created a Schiff base hydrogel by reacting chitosan with pyridine-based aldehyde and cross-linking it with epichlorohydrin. TEM characterizations were performed on the produced hydrogels. The TEM scans revealed spherical particles ranging in size from 25 nm to 64 nm. Nguyen and colleagues [46] developed an antibacterial chitosan/polyvinyl alcohol hydrogel containing silver nanoparticles for wound healing purposes. TEM examination was used to examine the structure and size of the hydrogels, as well as the presence of integrated silver nanoparticles in the hydrogels. El-Hady et al. [47] used a similar strategy to manufacture a curcumin/silver nanocomposite-based chitosan hydrogel, and the presence of silver nanoparticles in the chitosan hydrogel was observed via TEM analysis; Lall and colleagues [44] described colloidal hydrogels forming from chitosan–caseinate polyelectrolyte complexes. Polyelectrolyte hydrogels were formed by combining negatively charged caseinate with polycationic chitosan. The chitosan–caseinate hydrogel has a porous structure with the largest pore size being 200 nm, according to TEM analysis (Figure 12).

Under vacuum, samples were coated with gold for SEM examination, and hydrogel surfaces and cross-sections were photographed. SEM analysis can indicate chitosan hydrogel miscibility and polymer mix compatibility with hydrogel matrix constituents. By combining chitosan and pyridoxal 5-phosphate in an aqueous acetic acid solution, Craciun et al. 2022 [48] created a self-healing chitosan hydrogel. SEM methods were used to examine the morphological appearance of the produced hydrogels. The generated hydrogel has a microporous shape with pore dimensions ranging from 10 m to 50 m depending on the water concentration. By varying the solution concentration, the formation of micropores is varied due to the entrapment of water into the hydrogels. Similarly, Pomari et al. [49] created a chitosan hydrogel that was crosslinked with genipin-reinforced cellulose nanocrystals. SEM pictures of chitosan/cellulose nanocrystal hydrogel demonstrated an improved pore shape as cellulose nanocrystal concentration increased. A thermos-responsive physically cross-linked chitosan-based injectable hydrogel drug carrier was presented by Ahsan et al. 2020 [37,50]. The surface morphology was studied using SEM, and the resulting hydrogels had a dense and porous network structure. Stanzione et al. 2021 [51] created chitosan-based hydrogels that are thermosensitive and injectable. Transverse and longitudinal sections of the produced hydrogels revealed high porosity with open pore structure with pore size of around 92 m, potentially allowing nutrients to flow and catabolites to be released. Malik et al. 2021 [52] described -cyclodextrin chitosan-based hydrogels with pH-responsive characteristics that may be tuned. The hydrogels were created via free radical polymerization of -cyclodextrin, chitosan, and methacrylic acid, as well as N′ N′ methylenebis-acrylamide. The SEM image of these hydrogels revealed an uneven, somewhat porous surface with big wrinkles and cracks.

### 4.2. Functional Groups Analysis

To examine the chemical interactions between functional groups that comprise the hydrogel structure, spectroscopy approaches are often utilized. When making hydrogels, Fourier transform infrared spectroscopy (FTIR) is widely used to monitor the progress of the polymerization processes. Li et al. [53] created a chitosan-based hydrogel. The interaction between silver ion and amine groups was thought to be the most important factor in cross-linking chitosan and silver ion in the hydrogel network, which led to the improved mechanical characteristics of the final hydrogel. Boido et al. [54] created injectable highly permeable chitosan-based hydrogels for mesenchymal stem cell culture. When the temperature was raised from 0 °C to 37 °C, the hydrogel quickly gelled. The presence of functional groups in freeze-dried chitosan hydrogels was determined using the FT-IR spectroscopy approach. Mahmoodi et al. 2021 [55] proposed a graphene oxide–chitosan hydrogel for adsorptive removal of diclofenac from aqueous solutions using a simple mechanical mixing approach. The produced hydrogel material and its structural alterations were investigated using FT-IR spectroscopy. El-Hady et al. 2020 [47] created a chitosan-based, pH-sensitive, antibacterial hydrogel. Physical cross-linking was used to create chitosan and chitosan nanocomposite hydrogels. The hydrogels remained stable at room temperature for a long time. FT-IR research confirmed the functional groups of chitosan, as well as the encapsulated curcumin and silver nanoparticles. Yao et al. 2019 [56] used UV polymerization to create a chitosan-based hydrogel by grafting it with poly(acrylic acid) (PAA) and poly(hydroxyethyl methacrylate). The relevant stretching peaks of each comonomer functional unit were evaluated by FT-IR characterization to validate the successful polymerization.

### 4.3. Thermal Stability Analysis

The thermal stability of the produced hydrogels is determined by utilizing a thermogravimetric analysis device and increasing the temperature from room temperature to 600 °C at a rate of 10 °C per minute in an inert (Ar or N_2_) environment. Ahsan et al. 2020 [50] conducted a thermogravimetry investigation to detect the heat degradation of the produced hydrogel (TGA). The hydrogel breakdown at high temperatures indicates that the produced hydrogel is substantially cross-linked and very stable at high temperatures. Timur et al. 2018 [57] used TGA to assess the thermal characteristics of their generated aryl functionalities cross-linked chitosan-based hydrogel and discovered that chitosan derivative hydrogels are less thermally stable than chitosan hydrogels. Similarly, Vlasceanu et al. 2020 [58] used TGA to characterize the temperature-dependent weight loss of chitosan-based hydrogels, and the results revealed that the prepared graphene oxide-reinforced hydrogels were more stable due to the formation of the cross-linked network structure of chitosan, genipin, and graphene oxide. Aijaz et al. 2017 [59] created a chitosan polyacrylonitrile hydrogel by cross-linking chitosan with polyacrylonitrile using vapors. TGA was used to investigate the thermal behavior of the chitosan/polyacrylonitrile blend hydrogel and revealed that the thermal stability of the prepared hydrogels depends on the number of cross-linking networks in the hydrogel structure. Ghauri et al. 2021 [60] created biodegradable chitosan-based guar gum-polyvinylpyrrolidone cross-linked hydrogels. TGA was used to analyze the effect of crosslinker on the thermal stability of hydrogels with regard to temperature, and it was discovered that cross-linking had a favorable effect on hydrogel framework. A greater number of cross-linkers improves hydrogel heat stability.

### 4.4. Mechanical Resistance Analysis

Mechanical resistance of chitosan hydrogels is regarded as an important quality for their application in a variety of scenarios. Chitosan hydrogels can mimic human tissue and might be employed as scaffolds in medication delivery and biological applications. The chitosan hydrogel should have some mechanical resistance to maintain structural integrity. Chitosan hydrogel mechanical resistance is often assessed by cutting them into dumbbell shapes and examining their elongation and tension characteristics using a universal testing machine, while the thickness of the hydrogel is measured with a caliper. Furthermore, using a texturometer, the puncture test may be used to assess the mechanical resistance of hydrogel [61]. Budianto et al. 2019 [62] used a semi- and fully interpenetrating polymer network approach to create chitosan-poly(N-vinyl-pyrrolidone) hydrogels. The swelling and mechanical characteristics of the produced hydrogels were assessed using a universal tensile testing instrument. Postnova et al. 2022 [63] described DNA-Chitosan aerogels and hydrogel regeneration. To create a transparent homogenous hydrogel, native ds-DNA and chitosan were assembled without common phase separation and precipitation. The homogenous hydrogels created displayed exceptional mechanical strength and elasticity. Eivazzadeh-Keihan et al. 2021 [42] proposed a unique cross-linked terephthaloyl thiourea–chitosan hydrogel-based nanobiocomposite scaffold for antibacterial applications. Mechanical testing of the produced hydrogel nanocomposite scaffolds revealed an effective mechanical strength of 649.56 kPa.

### 4.5. Viscosity Testing

Sol-gel transition measurement is used to determine the hydrogel viscosity. The development of thermos-responsive chitosan-based hydrogel required this sol-gel transition test. When changing the temperature stimuli, these thermos-responsive hydrogels transform from the solution state to the gelation state. The swelling test is a quantitative parameter that is required to determine the amount of biological media or water that could be retained inside the hydrogel network. The dry hydrogel weight is measured as control and the dried sample is placed into water or biological media for about 48 h. Furthermore, the temperature variation is applied for the hydrogel, which is thermos-responsive. Finally, from the swelling test, the water-adsorbed hydrogel is measured to determine the amount of water taken up by the dried hydrogel. Khan et al. 2014 [64] studied the effect of viscosity and swelling behavior of the chitosan/polyvinyl alcohol hydrogels. Similarly, Aliouche et al. 2019 [65] studied the swelling kinetics and rheological behavior of chitosan–polyvinly alcohol/montmorillonite hybrid polymers and found that the prepared chitosan–polyvinyl alcohol hydrogel systems exhibited high swelling degrees and suitable viscoelastic properties.

### 4.6. Antimicrobial Properties of Chitosan Hydrogels

Chitosan has been proven to suppress the growth of bacteria, filamentous fungi, and yeast strains. Chitosan has also been discovered as an antibacterial agent, though its capacity to do so is uncertain due to the fact that its character has been attributed to several unique mechanisms. Chitosan has a broad spectrum of action and a high mortality rate against Gram-positive and Gram-negative bacteria. While Gram-negative microbes are mostly nontoxic to mammals, the molecular size of a substance is thought to determine its bactericidal properties. Hamedi et al. [66] investigated the antibacterial properties of six chitosan oligomers with widely differing molecular masses. To test this hypothesis, Gram-positive and Gram-negative microorganisms were used. They observed that chitosan greatly inhibited the growth of the majority of the bacteria tested; however, the inhibitory effects varied depending on molecular size and strain. Chitosan exhibited a greater bactericidal effect on Gram-positive bacteria than on Gram-negative bacteria.

## 5. Application of Chitosan-Based Hydrogels

Smart chitosan hydrogels respond to external stimuli and have become a focus of research in recent years. In general, smart chitosan hydrogels are classified as follows: hydrogels that are thermosensitive, photosensitive, or pH-sensitive [67]. Thermosensitive hydrogels are frequently used in biomedicine. At body temperature, this material passes through a sol-gel transition. Chitosan is thermosensitively modified by adding chemicals such as β-glycerophosphate and poloxamer to chitosan hydrogels. β-glycerophosphate is a common thermosensitive material that may thermally induce protons to move from chitosan to glycerophosphate, decreasing electrostatic repulsion and promoting hydrogen bond formation among chitosan chains, resulting in a sol-gel transition. Nguyen et al. [68] produced thermos-responsive injectable hydrogel via sol-gel transition at body temperature using integrated chitosan and oxidized cellulose nanofibers. The hydrogel showed the abilities of anti-inflammatory or wound healing (M2) macrophage 14 days after implantation.

Thermosensitive hydroxybutyl chitosan is a hydrogellic chitosan derivative that is frequently used in biomedical and medicinal applications. There are no organic cross-linking agents. This biocompatible and reversibly thermo-responsive hydrogel requires sol-gel transition. pH-sensitive hydrogels are hydrogels whose dimensions change depending on the pH of the surrounding environment. The pH of the damaged region changes during the healing process. During the wound-healing process, the expansion of chitosan hydrogels can speed up cell infiltration and proliferation while also facilitating healing. Osmosis of oxygen, a pH-sensitive chitosan methacrylate hydrogel with tunable mechanical characteristics and swelling ratio, was created by Zhu et al. [69] based on pH changes during wound healing. This smart hydrogel swells at acidic pH and deswells at physiological pH; therefore, it can be applicable on the release of anti-inflammatory drugs during the initial wound healing treatment.

### 5.1. Antibacterial Hydrogels for Drug Delivery 

**Figure 13:**
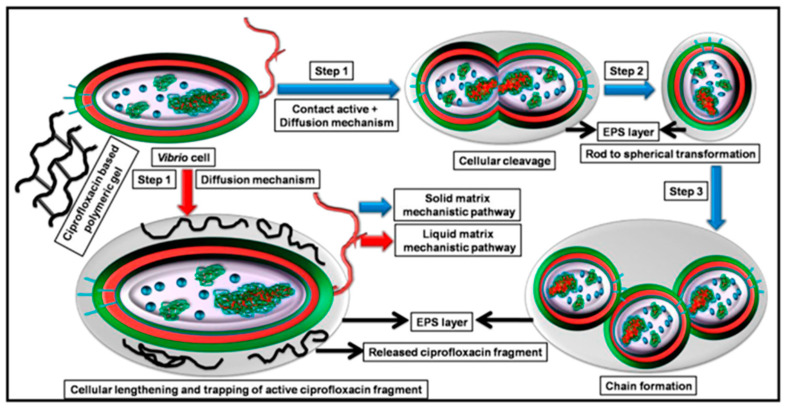
The bactericidal mechanism of DCHBSP gel (ciprofloxacin-based polymeric gel) against *V. chemaguriensis* strains in solid and liquid matrices. Adopted from open access resource [26].

The most significant threats to human health are infectious diseases caused by harmful microorganisms. According to a World Health Organization (WHO) study, they are the world’s second leading cause of death. As a result, efficient antibacterial materials that are multifunctional and easy to fabricate are required. Antibacterial hydrogels are among the materials with high mechanical characteristics that might be used in a variety of biomedical applications [70]. Hydrogels are some of the most suitable biological materials for drug delivery in the field of antibacterial treatment. The antibacterial properties of the hydrogels were investigated using various methods, including the minimum inhibition concentration method (MIC), the zone of inhibition method (disc diffusion and well diffusion), the oscillation culture method, and scanning electron microscopy (SEM), among others. There are two types of anti-microbial hydrogels: intrinsic or self-antibacterial hydrogels and loaded anti-bacterial hydrogels. Guo et al. developed an in situ formation of hydrogel using N-carboxyethyl chitosan and dibenzaldehyde-terminated polyethylene glycol, and applied it as a drug delivery carrier for the delivery of doxorubicin drug by employing the pH-responsive Schiff base. This hydrogels exhibited in vitro pH-dependent gel-degradation-based drug release [71]. There are several types of self-antibacterial hydrogels, such as peptide-based hydrogels, chitosan (CTS)-derived hydrogels, dextranaldehyde/polyethyleneimine hydrogels and inorganic composite hydrogels. Zhang et al. [72] prepared an injectable and temperature-sensitive chitosan-based hydrogel prepared using hyaluronic acid and sodium glycerophosphate for pH-sensitive drug delivery with cell adhesive properties. Furthermore, the loaded antibacterial hydrogels comprised inorganic nanoparticles, antibiotic-loaded antibacterial hydrogel, photosensitive antibacterial hydrogel, and hydrogels with synergetic effects [26] (Figure 13).

### 5.2. Antibacterial Hydrogels for Wound Dressing 

**Figure 14:**
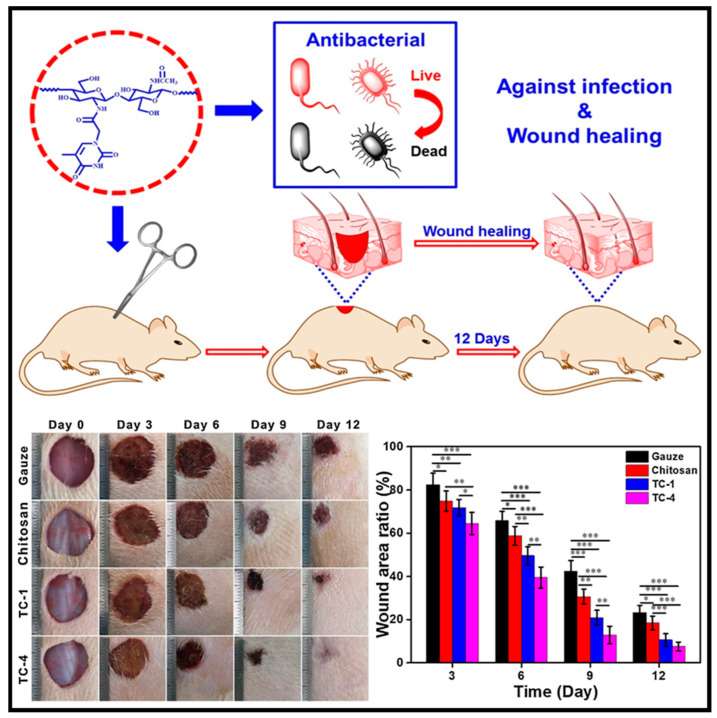
This figure represents the application of hydrogels against infected wounds on mice model. Representational photographs of the healing progress of the wounds covered with gauze, and the sponges of chitosan, adopted from [78] with permission from Elsevier through copyright clearance center.

The current focus of antibacterial hydrogel research is on the development of hydrogels consisting of naturally derived antibacterial polymers. Chitosan (CTS) is an appealing material for use in healthcare due to its biocompatibility and antibacterial properties. Chitosan is a cationic antibacterial polymer. Chitosan naturally contains antibacterial properties that inhibit the growth of bacteria and fungi. Zhang et al. 2021 [73] investigated the carboxymethyl chitosan-based hydrogel film for antibacterial wound dressing and described a good injectable chitosan hydrogel system with antibacterial activity, biocompatibility, and self-repairing capabilities. The skin plays a crucial role as the physical barrier between the surrounding environment and the physiological body. Burns, infections, and acute and chronic skin disorders such as psoriasis are all examples of skin damage. The hydrogel film presented significant antibacterial activity against *Escherichia coli* (*E. coli*) and *Staphylococcus aureus* (*S. aureus*). Therefore, the hydrogel films with high mechanical strength and high antibacterial activity could be used in wound dressing applications. Many researchers developed chitosan-based hydrogel. Wangi et al. [74] developed an injectable chitosan-based self-healing hydrogel system using chitosan and aldehyde-modified chitosan via dynamic Schiff base reaction. The results showed that the prepared hydrogel has good rheological properties, injectability, self-healing properties suitable in antibacterial-based wound healing applications. The chitosan hydrogels inhibited the growth of pathogenic bacterial cultures such as *E. coli*, *S. aureus*, *P. aeruginosa*, and *C. albicans* (Figure 14) [36,39,72,73,74,75,76,77,78,79].

### 5.3. Antibacterial Hydrogels for Tissue Engineering

In the last decade, several investigations have focused on chitosan-based hydrogels for tissue engineering. Chitosan’s biodegradable, biocompatible, and nontoxic features, among other natural polymers, support its selection due to its antibacterial, bio adhesive, biological activity, and hemostatic effects [12]. The use of HGs in tissue engineering is useful because they can organize cells in three dimensions, provide optimal mechanical integrity to build new tissues, and allow nutrition transport to encapsulated cells [80]. Recently, Chen et al. [81] prepared a thermos-responsive hydrogel using chitosan/gelatin for therapeutic angiogenesis. They found that the gelatin blending with hydrogel provides an appropriate microenvironment for adipose-derived stem cells, demonstrating that the developed hydrogel system is suitable to accelerate ischemic tissue regeneration. 

Liang et al. [82] developed polyelectrolyte-based homogeneous composite hydrogel scaffold using chitosan and carrageenan via a cross-linking method using epichlorohydrin crosslinker. In vitro results of the prepared hydrogel system showed bio adhesion, biocompatibility, and proliferation of ATDC5 cells suggesting that this hydrogel is promising for cartilage repair. Wang et al. [80] created new scaffolds from hydroxyethyl chitosan (HECS) and cellulose (CEL), using chemical cross-linking, silica leaching, and freeze-drying methods to create micro- and macropores via a particulate porogen and freeze-drying process. In vitro bioanalysis revealed that HECS/CEL scaffolds could promote the attachment, spreading, viability, and proliferation of osteoblastic MC3T3-E1 cells, making them a suitable matrix for use in bone tissue development.

### 5.4. Chitosan Based Hydrogels for Antifungal and Antibiofilm Activity

Hydrogels have lately acquired popularity as good antibacterial compositions that may be used in a variety of biological applications. Antimicrobial hydrogels are hydrated polymeric networks that have the ability to eliminate pathogenic microorganisms and have been utilized in a variety of pharmaceutical applications, including medications, disinfectants, sanitizers, and personal care products [83]. Hydrogels displayed antibacterial activity after incorporating antimicrobial chemicals or cationic polymers into their networks. After incorporating antibacterial chemicals or cationic polymers into their networks, hydrogels demonstrated antibacterial activity. Antimicrobial hydrogels have been classified into two groups depending on their antimicrobial activity source: hydrogels utilized as a matrix for antimicrobial agents (e.g., Ag nanoparticles, antibiotics, chemical compounds, and so on) and hydrogels with intrinsic antimicrobial activity (e.g., natural polymer-based hydrogels, synthetic polymeric hydrogels, peptide-based hydrogels, etc.) (Table 1).

Bacterial biofilm development on the surface of bodily tissues and medical equipment is common in chronic and recurring infections [37]. Biofilms are a well-organized community of bacterial cells created by a wide variety of bacteria, and they have been linked to significant diseases [84]. According to the National Institute of Health (NIH), biofilms are responsible for up to 65% of microbial infections and more than 80% of serious diseases [85]. Biofilms are a worldwide public health problem due to their ability to grow on both biotic and abiotic surfaces, resulting in chronic, recurrent, and nosocomial bacterial infections. As a result of a lack of self-cleaning, bacterial coatings populate these medical devices, making them susceptible to contamination during common usage or the implantation procedure [86]. Furthermore, the treatment cost of biofilm-related disorders is estimated to be $94 billion, with an economic loss of $11 billion observed in the United States alone as a result of biofilms’ effect on the health sector [87].

**Figure 15:**
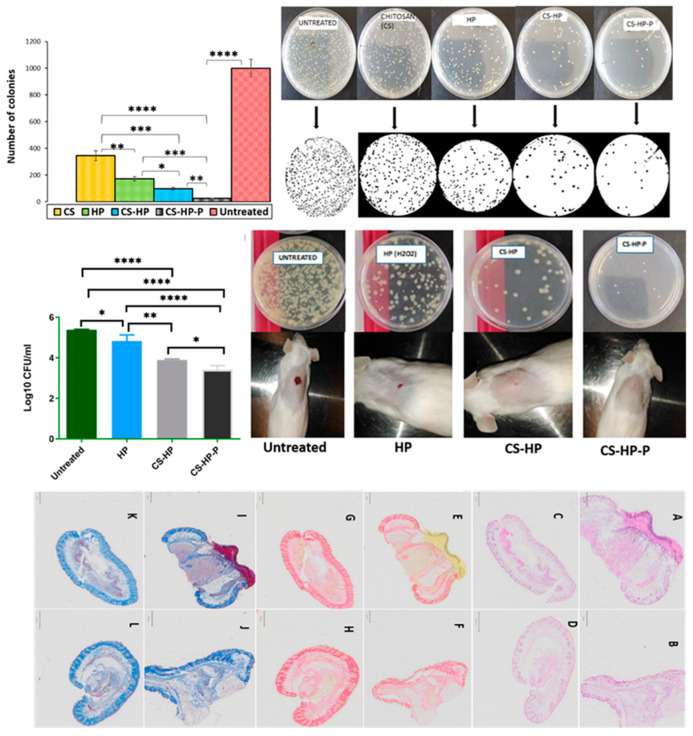
Number of colonies of untreated MRSA biofilms and biofilms exposed by chitosan-based hydrogels. Evaluation of MRSA burden post-treatment. Physical observation of the wound closure and bacterial burden. Photomicrographs of the untreated and treated skin sections stained with H&E, PSR, and CAB (0.5×), adopted from open access resource [39].

Wound healing is characterized by highly coordinated, conserved, and spatiotemporally regulated processes, as well as sequential phases such as hemostasis, inflammation, proliferation, and remodeling (Figure 15). Bacterial biofilm colonies have been seen in over 60% of chronic wounds [70]. A biofilm is formed when a colony of bacteria is enclosed in a polymer matrix that functions as a mechanical barrier to immune system cells and antibiotics, allowing the bacteria to thrive. Biofilm control has become an important aspect of wound treatment. Biofilms impair wound healing by generating bacterial infection, inflammation, delayed healing, decreased fibroblast activity, decreased angiogenesis, and collateral tissue damage [88]. 

The advent of antibiotic-resistant bacteria strains has confirmed the use of substantial doses of antibiotics in the treatment of biofilms. The main mechanism that leads to tolerance and resistance to antimicrobial agents by biofilms is the development of nutrient and oxygen gradients that slow the penetration of antibiotics to the core of the biofilm as a result of starvation caused by the high density accumulation of bacteria in a specific location [89]. Biofilms are often made up of complex groups of living microorganisms attached to a layer and surrounded by a self-producing extracellular matrix comprising polysaccharides, extracellular DNA, and proteins. As a result, the biofilm issue complicates the antimicrobial drug resistance situation.

### 5.5. Chitosan-Based Hydrogels for Antifungal Applications 

Fungal invasive infections are becoming more common as the number of immunocompromised people grows, with an estimated attributable global mortality of more than 1.5 million deaths per year [90,91,92]. The mucocutaneous tissues (oral or vulvovaginal mucosa) are commonly affected by *Candida* species’ superficial mycotic infections [39,93,94,95]. As a result, the challenge over the last several decades has been to create unique treatment techniques for candidiasis therapy from the beginning, in order to minimize fungal spread in the circulation and internal organs [96]. Because of their distinct physical properties, hydrogels are ideal for integrating, transporting, and delivering therapeutics and other biologically active substances [97]. Because of their ability to absorb large amounts of water and their elasticity, hydrogels are similar to biological tissues. Hydrogels may be generated by physical or chemical cross-linking and are an effective drug delivery strategy in the treatment of fungal infections. Hydrogels have unique biological features, such as biocompatibility and biodegradability, and a variety of polysaccharides are used in hydrogel-based therapeutic formulations. Among them, chitosan-based polysaccharides are gaining popularity in the development of a wide range of hydrogels. The copolymers of glucosamine and N-acetylglucosamine units connected by b-1,4-glycosidic linkages (-NH_2_ at C_2_ and -OH at C_3_ and C_6_) in the chains provide both chemical reactivity and bioactivity to the chitosan-based hydrogels. Chitosan has a high potential for use as an antifungal agent in the treatment of diseases caused by human pathogenic fungi. Chitosan causes energy-dependent permeabilization of plasma membranes in sensitive fungi. Chitosan causes energy-dependent permeabilization of plasma membranes in sensitive fungi. This polymer has an antibacterial effect against harmful microorganisms. Chitosan suppresses the production of biofilms in *A. fumigatus* [97].

Lo et al. 2020 [98] reported that six commercial chitosans with varied molecular weights and degrees of deacetylation were investigated for antifungal efficiency and synergistic effects with antifungal drugs against *Candida albicans* SC5314, *Candida tropicalis* MYA3404 and drug-resistant strains. Heedy et al. 2020 [99] used solvent-assisted drop cast lithography to create arrays of varying diameter nanopillars for surface coating with the microbiostatic biopolymer chitosan, which has excellent antibacterial properties against fungi and bacteria and is biocompatible with human epithelial cells. According to the research, the surface inhibits the growth of dangerous fungi and bacteria. When compared to a control, the bacteria count on the flat surface decreased, demonstrating that the surface is naturally antibacterial.

Hydrogels are often used in dermatology because they are emollient, easily removable, have optimal spread ability, higher compatibility, are water-soluble, and are pseudo-plastic [100]. Chouhan et al. 2022 [101] developed a nickel-conjugated chitosan hydrogel to treat Fusarium rot of wheat, and their results showed that pathogenic stress tolerance capability improved seedling vigor. He and coworkers [102] prepared a chitosan-Ag hydrogel by physical cross-linking approach and the effect of prepared chitosan-Ag hydrogel against the fungal species during grape storage were evaluated. Their results showed that the chitosan-Ag hydrogel showed a fungal inhibitory effect and a positive fresh-keeping effect. 

The ongoing search for molecules with intrinsic antimicrobial activity is critical for both the prevention and treatment of *Candida* infections. Chitosan is a naturally occurring polysaccharide biopolymer with remarkable biological features, such as non-toxicity, biodegradability, and biocompatibility, making it a great choice for biomedical applications [103,104,105,106,107]. Chitosan also has antibacterial action against a variety of microorganisms, including algae, fungi, and bacteria. Furthermore, it was demonstrated that chitosan treatment may effectively minimize or prevent bacterial biofilm formation in vitro and in vivo.

## 6. Conclusions and Future Prospect

In this review, we focused on the preparation of chitosan-based hydrogels, characterization techniques, and their antimicrobial and antibiofilm applications. In the past few decades, the development of hydrogel-based scaffold materials has been a milestone in the area of antimicrobial and antibiofilm applications. However, the development of biopolymer-based hydrogel materials still needs to be explored. The physical and chemical properties of the hydrogel-based scaffold materials should be improved when applying them in various biological applications, including antimicrobial- and antibiofilm-based applications. Antibacterial hydrogels have a wide range of applications, including wound dressings, urinary tract coatings, catheter-associated infections, gastrointestinal infections, osteomyelitis, and contact lens use. Nonetheless, various issues with chitosan-based hydrogels must be addressed. For example, chitosan’s weak solubility and hydrogels’ poor mechanical characteristics limit its use in medical devices. Components such as antibiotics, nanoparticles, and other substances placed into the hydrogels provide them antibacterial properties. Further research will be required to determine how to increase the solubility of chitosan materials in order for them to be conveniently performed or shaped, to reduce the toxicity, and to maximize all advantages while retaining their inherent properties. We look forward to the development of multifunctional chitosan hydrogels with promising antimicrobial therapy applications. Finally, despite the fact that chitosan particle systems have been explored for more than two decades, with consistently promising findings in the administration of various therapeutic agents, both in vitro and in vivo in experimental animals, no commercial formulations of these systems are available. This might be due to inadequate bioavailability, potential immunogenicity and long-term toxicity, low target specificity, limited stability, and a lack of human clinical studies, all of which require more investigation.

## Figures and Tables

**Table 1:** Different types of chitosan-based hydrogels for antimicrobial activity.

Antimicrobial Activity	Hydrogel Type	References
Antibacterial activity against *Escherichia coli* and *Staphylococcus aureus*	Carboxymethyl chitosan (CMCS)	[73]
*E. coli* ATCC 25922, *S. aureus* ATCC 6583 and *Candida albicans* ATCC 10231	Chitosan-based Hydrogels	[74]
Gram-positive bacteria (*S. aureus*), Gram-negative bacteria (*E. coli*), *C. albicans* and methicillin-resistant *S. aureus* (MRSA)	Chitosan-based hydrogels	[83]
Two pathogenic strains including Gram-negative (*Pseudomonas aeruginosa*) and Gram-positive (*S. aureus*)	Carboxylated chitosan	[76]
*E. coli* and *S. aureus*	Chitosan-based hydrogel	[75]
Gram-positive bacteria (*S. aureus*), Gram-negative bacteria (*E. coli*), *C. albicans*, MRSA, *P. aeruginosa* and *Acinetobacter baumannii*	Chitosan-based hydrogel	[37]
*E. coli* and *S. aureus*	Polyvinyl alcohol (PVA)/N–succinyl chitosan (NSCS)/lincomycin hydrogels	[79]
*E. coli* and *S. aureus*	Chitosan hydrogel	[36]
MRSA bacteria according to a protocol that has been reported	Chitosan-based Hydrogel	[39]
*E. coli*, *S. aureus*, *P. aeruginosa*	Chitosan-based Hydrogel	[80]
*E. coli*	Chitosan-based Hydrogel	[80]

## Data Availability

Not applicable.
